# Improvements in HOMA indices and pancreatic endocrinal tissues in type 2-diabetic rats by DPP-4 inhibition and antioxidant potential of an ethanol fruit extract of *Withania* *coagulans*

**DOI:** 10.1186/s12986-021-00547-2

**Published:** 2021-04-21

**Authors:** Heera Ram, Pramod Kumar, Ashok Purohit, Priya Kashyap, Suresh Kumar, Shivani Kumar, Garima Singh, Abdulaziz A. Alqarawi, Abeer Hashem, Elsayed Fathi Abd-Allah, Al-Bandari Fahad Al-Arjani, Bhim Pratap Singh

**Affiliations:** 1 Department of Zoology, Jai Narain Vyas University, Jodhpur, Rajasthan 342001 India; 2 University School of Biotechnology, Guru Gobind Singh Indraprastha University, Dwarka, Sector 16C, New Delhi, India; 3 Department of Botany, Pachhunga University College, Aizawl, Mizoram 796001 India; 4 Plant Production Department, College of Food and Agricultural Sciences, King Saud University, P.O. Box. 2460, Riyadh, 11451 Saudi Arabia; 5 Botany and Microbiology Department, College of Science, King Saud University, P.O. Box. 2460, Riyadh, 11451 Saudi Arabia; 6 Mycology and Plant Disease Survey Department, Plant Pathology Research Institute, ARC, Giza, 12511 Egypt; 7 Department of Agriculture and Environmental Sciences (AES), National Institute of Food Technology Entrepreneurship and Management (NIFTEM), Sonepat, 131028 Haryana India

**Keywords:** HOMA, β-Cells, Pancreatic histology, Antioxidants, DPP-4, Phytochemicals

## Abstract

**Context:**

*Withania coagulans* (Stocks) Dunal fruits are used in the therapeutics of several ailments due to possessing of potent phytoconstituents which is also used traditionally for curing the diabetes.

**Objective:**

The present study was assessing the amelioration potential of the phytochemicals of an ethanol fruit extract of *W.* *coagulans* (Stocks) Dunal in the HOMA (Homeostatic model assessment) indices and pancreatic endocrinal tissues by inhibition of DPP-4 and antioxidants activities.

**Material and methods:**

The identification of phytoconstituents of the test extract was performed by LCMS. Further, assessments of in-vitro, in-vivo and in-silico were achieved by following standard methods. In-vivo studies were conducted on type-2 diabetic rats.

**Results:**

The chosen extract inhibited DPP-4 activity by 63.2% in an in vitro assay as well as significantly inhibit serum DPP-4 levels. Accordingly, the administration of the ethanol fruit extract resulted in a significant (*P* ≤ 0.001) alterations in the lipid profile, antioxidant levels, and HOMA indices. Moreover, pancreatic endocrinal tissues (islet of Langerhans) appeared to have the restoration of normal histoarchitecture as evidenced by increased cellular mass. Molecular docking (Protein-ligands) of identified phytoconstituents with DPP-4 (target enzyme) shown incredibly low binding energy (Kcal/mol) as required for ideal interactions. ADMET analysis of the pharmacokinetics of the identified phytoconstituents indicated an ideal profile as per Lipinski laws.

**Conclusion:**

It can be concluded that the phytoconstituents of an ethanol fruit extract of *W.* *coagulans* have the potential to inhibit DPP-4 which result in improved glucose homeostasis and restoration of pancreatic endocrinal tissues in type-2 diabetic rats.

## Introduction

Diabetes Mellitus is chronic and complex metabolic disorder in which the role of the DPP-4 enzyme has been established. DPP4 rapidly degrades GLP-1(glucagon like Peptide-1) and plays a crucial role in glucose homeostasis [1]. DPP4 inhibitors block the degradation of GLP-1, the latter of which is responsible for stimulating insulin secretion, and thus plays a significant role in regulating glucose homeostasis [2]. The present study assessed the antidiabetic potential of an ethanol fruit extract of *Withania* *coagulans.* The use of herbal medicines based on historical knowledge has gained greater acceptance throughout the world [3]. The use of plants in herbal medicine represents a reservoir of historic information that has been developed over countless generations [4, 5]. The Ayurvedic medicine, traditional Chinese medicine (TCM) and integrative medicine represent a significant Asian legacy based on thousands of years of research and healthcare [6].

Information from several studies suggest that the various phytoconstituents present in plants, such as the flavonoids, saponins, tannins, alkaloids, glycosides, and terpenes, possesses anti-diabetic properties [7]. The anti-diabetic effect of the phytocompounds has been proposed to be based on several mechanisms working alone or in parallel, including stimulation of insulin secretion, reduction in hepatic glucose uptake, inhibition of enzymes involved in carbohydrate metabolism (such as α-glucosidase inhibitors), modulation of molecules such as PPARγ, hypolipidemic action, antioxidant potential, interference with the action of glycolytic enzymes (such as phosphoenolpyruvate), carboxykinase activity, and augmentation of the expression of glucose transporters, etc. [8].

In this regard, the fruit of *W.* *coagulans*has gained interest for its antidiabetic activity in some animal models, as well as in pilot trials in humans [9–11]. *W. coagulans* fruit possesses a variety of bioactive phytoconstituents that vary in their polarity, solubility, and specific chemical and physical properties [11]. Phytochemical studies have reported that the main phytoconstituents of the fruit are esterases, free amino acids, fatty oils, essential oils, and withanolides [12]. The withanolides, which are steroidal lactones with an ergostane skeleton, represent the predominant phytoconstituents present in *W. coagulans* fruit [13].

Previous studies have reported that *W. coagulans* fruit has been used for a variety ethnomedicinal uses, including anti-inflammatory, cardiotonic activity, hepatoprotective, antifungal, hypoglycemic, free-radical scavenging activity, hypolipidemic, wound healing activity, and for the treatment of diabetic nephropathy [9]. Extracts obtained from different parts of *W. coagulans* fruit contain a different profile of phytoconstituents. Notably, a systematic in-vitro, in-vivo*,* and in-silico analyses of the specific phytoconstituents present in an ethanol extract of *W. coagulans* fruit has not been conducted. Therefore, the objective of the present study was to evaluate the ability of an ethanol extract of *W. coagulans* fruit to maintain glucose homeostasis and restore the histology of endocrinal pancreatic tissues in type 2 diabetic rats through its inhibitory effect on DPP-4 and its antioxidant potential.

## Material and methods

### Experimental design

The experimental design was formulated in comparison to the control and treated groups where each group consisting of six wistar rats (*Rattus norvegicus*) (n = 6) with twice repeated schedule. The treatments were performed by oral administration for four weeks and these groups were compared to the vehicle (non-treated, normal metabolism) and diabetic control groups. The treated groups were received the ethanol fruit extract of *W. coagulans* and the standard diabetic drug, sitagliptin. The protocols used in the animal experiments were approved by the IAEC (Institutional Animal Ethical Committee) as per norms of the CPCSEA (Committee for the Purpose of Control and Supervision of Experiments on Animals), Government of India (Reg. No.1646/GO/a/12/CPCSEA valid up to 27.03.23).

### Induction of type-2 diabetes

Type-2 diabetes was induced in the rats through administration of a high-sucrose diet along with high-carbohydrate food for three weeks. Four intraperitoneal injections of dexamethasone (1.0 mg/kg) at alternate day intervals was also used to induce type 2-diabetes in the rats by following modified protocol [14]. The establishment of type-2 diabetes in the rats was determined by monitoring the levels of glucose and insulin through HOMA (Homeostasis Model Assessment) indices (HOMA-IR (insulin resistance), HOMA-β% (β-cell function), and HOMA-S% (insulin sensitivity) [15] (Additional file 1).

### Fruit extract, standard drug, and chemical reagents

The ethanol fruit extract of *W.* *coagulans* was prepared using a standard Soxhlet protocol. The obtained extract was subsequently evaporated to dryness in a vacuum and the dried powder was used to formulate the extract [16]. The sitagliptin (Januvia® 50 mg), was purchased from a local pharmacy in Jodhpur, India. The dose of extract (400 mg/kg) was provided to the treated rats as per calculations of physiological dose [17]. Chemical reagents were purchased from a local supplier and were of a chemical grade equal to Loba Chemie Pvt Ltd. Biomedical diagnostic kits (Erba, Pvt Ltd) were used for the biochemical analysis of blood serum and DPP-4 inhibition assay kit (Sigma Aldrich) was used for the DPP-4 inhibition assay.

### Identification of the phytoconstituents present in the ethanol extract of *W. coagulans* fruit by LC–MS analysis

The phytoconstituents present in the ethanol extracts of *W. coagulans* fruit were identified by LC–MS (Liquid chromatography and Mass spectroscopy) analysis using standard protocols [18]. The LC–MS analysis was outsourced to CDRI (Central Drug Research Institute), Lucknow, India and performed by trained technicians on the appropriate equipment (ID: FEE-2, SAIF920). The HPLC samples were further analysed by Q-TOF mass spectrometry equipped with an ESI source. The analysis conditions were as follows: full-scan mode from m/z 50 to 1200 and a source temperature of 140 °C. The solvent was methanol with 0.3% formic acid. Solvents were subjected to a flow rate of 0.1 mL/min. The MS spectra were acquired in the positive ion mode. The mass fragmentations were identified using the spectrum database and mass hunter software.

### Inhibition of DPP-4 activity and treatment of hyperglycemia

Two groups of rats were used to assess the impact of treatments on type 2- diabetic rats. The ethanol extract of *W.* *coagulans* fruit and the standard drug, sitagliptin, were the two assessment treatments groups. Group-III (WCEt) the formulated fruit extract at a dose of 400 mg/kg BW (Body Weight) per day was administered to type -2 diabetic rats [19]. Group -IV (SITA) sitagliptin at a dose of 50 mg/kg body weight per day, which is equivalent to a 50 mg oral clinical dose, was administered to another group of type 2-diabetic rats. Group-I (VC) and Group II (DC) rats were served as negative and positive controls, respectively. The extract and drug administration were performed by gastric intubation between 10 and 11 AM to avoid variable responses due to circadian rhythms.

### In-vitro and in-vivo (serum) inhibition assay of DPP-4

The in-vitro DPP-4 assay was performed using the standard protocol of measuring chromatophore production by the cleavage of Gly-Pro p-nitroanilide hydrochloride. The inhibition of DPP-4 by the phytoconstituents of fruit extract was determined by measuring the release of 4-nitroaniline from an assay mixture that included 0.1 M Tris–HCl (pH 8.0) and 2 mM Gly-Pro p-nitroanilide (substrate). The reaction mixture was incubated at 37 °C and moderated by the addition of sodium acetate buffer (pH 4.5). Absorbance was measured at 405 nm using a UV–VIS Spectrophotometer [20, 21]. Percent inhibition was calculated using the following formula.

$${\text{\% }}\,{\text{inhibition}} = \frac{{{\text{Absorbance}}\,{\text{of}}\,{\text{control-Absorbance}}\,{\text{of}}\,{\text{inhibitor}}}}{{{\text{Absorbance}}\,{\text{of}}\,{\text{Control}}}} \times 100$$

Accordingly, the serum DPP-4 assay was performed by following the above mentioned protocol with replacement of test sample of WC fruit by 10 μL of serums from the experiment groups as reported by earlier studies [22, 23].

### Biochemical analysis of blood serum

(a) Basic parameters: The serum parameters measured by using standard methods included glucose [24], total protein [25], insulin [26], total cholesterol [27], HDL-cholesterol [28], triglyceride [29], SGOT [30], SGPT [30], urea [31], uric acid [32], and creatinine [33]. The lipid profile (total cholesterol, HDL-cholesterol, LDL-cholesterol, Triglyceride and VLDL-cholesterol) was assessed following Friedewald’s formula [34–36].

$${\text{LDL-C}}\,\left( {{\text{mg}}/{\text{dL}}} \right) = {\text{TC}}\,\left( {{\text{mg}}/{\text{dL}}} \right) - {\text{HDL-C}}\,\left( {{\text{mg}}/{\text{dL}}} \right) - {\text{TG}}\,\left( {{\text{mg}}/{\text{dL}}} \right)/5.$$

(b) Total antioxidant capacity (FRAP) [37], catalase [38], SOD [39], GSH [40], and LPO activity [41] were assessed by following the standard methods.

### HOMA (Homeostatic model assessment) analysis

(a) (HOMA-IR and HOMA-β) scores and insulin sensitivity were determined using fasting serum insulin and glucose concentrations measured at the end of the experiment. Calculations were based on the formula reported by Matthew et al. and Parekh et al. as follows [42, 43].

$${\text{HOMA-IR }} = \frac{{{\text{Fasting}}\,{\text{Insulin}}\,\left( {{\text{U}}/{\text{L}}} \right) \times {\text{Fasting}}\,{\text{Glucose}}\,\left( {{\text{mmol}}/{\text{L}}} \right)}}{22.5}$$

$${\text{HOMA}} -\upbeta = \frac{{20 \times {\text{Fasting}}\,{\text{Insulin}}\,\left( {{\text{U}}/{\text{L}}} \right)}}{{{\text{Fasting}}\,{\text{Glucose}}\,\left( {{\text{mmol }}/{\text{L}}} \right)}} - 3.5$$

$${\text{Insulin}}\,{\text{sensitivity}}\,\left( {{\text{IS}}} \right) = \frac{1}{{\left[ {{\text{Insulin }}\left( {\frac{{\text{U}}}{{\text{L}}}} \right) \times {\text{Log }}\left( {{\text{glucose}}\,\left( {{\text{mmol}}/{\text{L}}} \right)} \right)} \right]}}$$

### Histopathology

Pancreatic tissues were obtained from autopsied animals after the completion of the experiments and processed for histological examination using standard methods [44]. Briefly, tissues were fixed in 10% formalin, gradually dehydrated in an ethanol series, and embedded in paraffin wax. The embedded tissues were sectioned at a 5-μm thickness, stained with hematoxylin and eosin, and were then subsequently observed with a clinical microscope and photomicrographs were taken with an attached camera.

### Molecular Docking analysis

The phytoconstituents identified by LC–MS analysis and the protein ligand molecular docking with the DPP-4 protein was assessed [45, 46]. Molecular interactions of the identified compounds with DPP-4 were investigated using PyMol and Autodock 4.2. The catalytic triad of DPP-4 comprises Glu205, Glu206, and Tyr226 as the main residues and a hydrophobic core is composed of ten residues (Tyr547, Tyr667, Asn710, Val711, His740, Ser630, Ser209, Arg358, Phe357, and Val207). A high-resolution crystallographic structure of DPP-4 receptor protein (PDB ID 5y7k) was downloaded from a public protein database and processed using PyMol to extract the co-crystallised ligand inhibitor, remove water molecules, and correct the chain integration. Three-dimensional structures of the identified compounds sitagliptin, and vildagliptin (two standard drugs with DPP-4 inhibitory activity) were downloaded from the Pubchem Database. Ligands were processed using PyMol and hydrogen was added to the structures. The developed docking protocol was validated by performing re-docking with prepared co-crystalized ligand and receptor protein and maps were generated. Post-validation was conducted of the docking protocol of the individual identified compounds with DPP-4 protein. Molecular interactions, ligand conformations, and binding energies for each of the phytoconstituents and the standard drugs were obtained.

### ADMET analysis

ADME/T (Absorption, distribution, Metabolism, Excretion, and Toxicity) analysis was performed using Drulito software (www.niper.gov.in/pi_dev_tools/DruLiToWeb/DruLiTo_index.html) to study the pharmacokinetics profile of the identified compounds for potential drug development [47, 48]. The compounds were ranked based on two filters: the Lipinski rule and the ability to pass through the blood brain barrier (BBB). The Lipinski rule states that an ideal drug molecule should weigh below 500 g/mol, hydrogen bond donors should be ≤ 5, and the number of hydrogen bond acceptors should be ≤ 10 and have a partition coefficient ≤ 5. A compound with these properties would pass the BBB if the number of hydrogen bonds present is between 8 and 10 and no acidic groups are present in the molecule. TPSA (total polar surface area) indicates the bioavailability of the drug molecule as per Veber’s rule. ATPSA ≤ 140 Å indicates good oral bioavailability.

### Statistical analysis

Values obtained for the biochemical assessments and other data were expressed as a mean ± the standard error of mean (SEM) and the effect of treatment was analyzed by a one-way ANOVA with a post hoc Dunnett’s *t*-test using SPSS 22 trial version for windows [49]. The probability of significant differences between treatment means was set at *P* ≤ 0.05.

## Results

Assessments of the in-vitro, in-silico*,* and in-vivo activity of the fruit extract in comparison to standard diabetic drugs and relevant controls were conducted. The identification of the major phytoconstituents present in the ethanol fruit extract of *W.* *coagulans* was also determined by phytochemical assessments of LCMS.

### In-vitro inhibition of DPP-4 activity

**Fig. 1:**
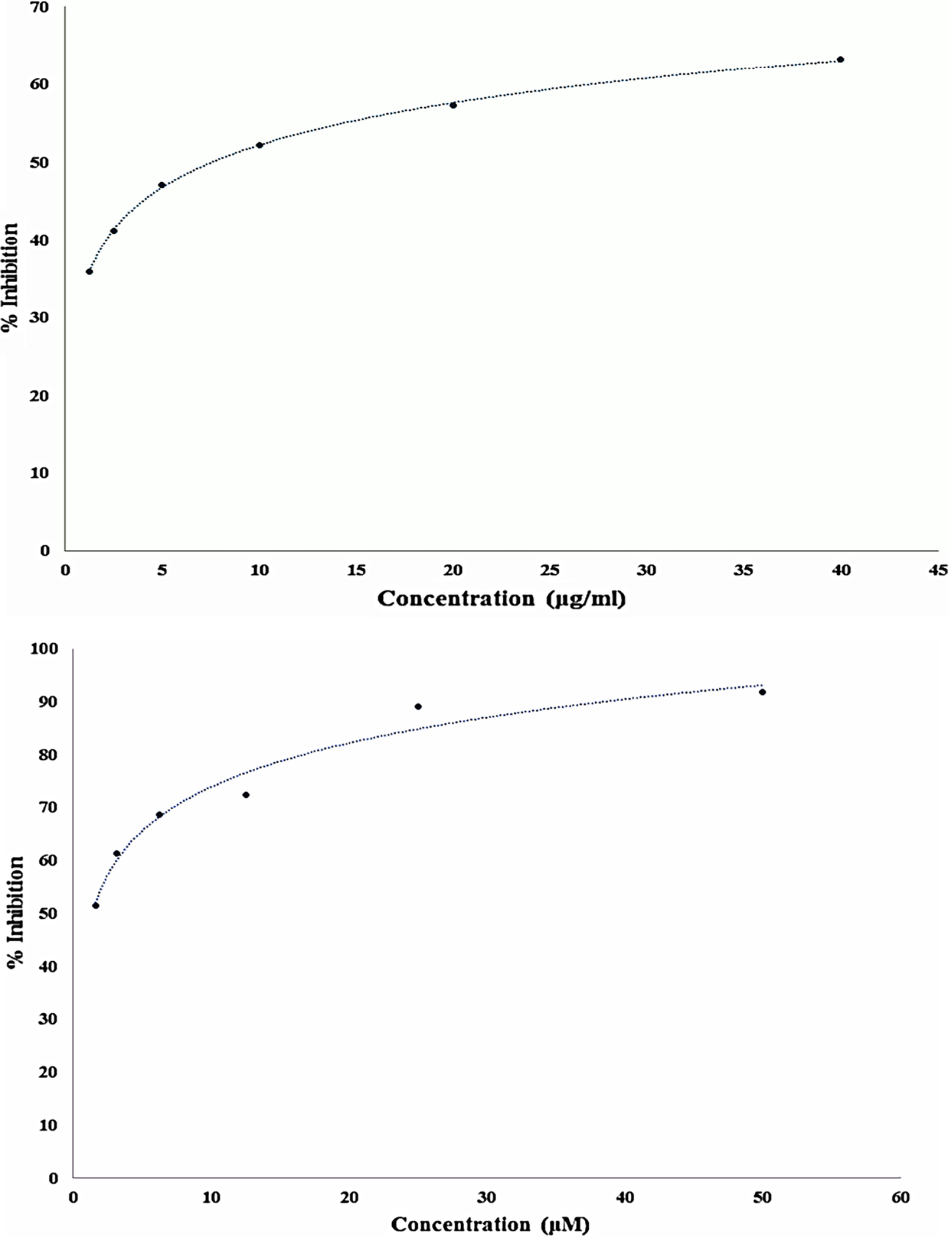
**a** In-vitro DPP-4 inhibition assay against ethanolic fruit extract of ethanol fruit extract of *Withania* *coagulans* (Equation- y = 7.8441ln(x) + 34.107, R^2^ = 0.9995, IC50 = 7.58 μg/ml). **b** In-vitro DPP-4 inhibition assay against sitagliptin (Equation- y = 11.953ln(x) + 46.305, R^2^ = 0.9671, IC50 = 1.36 μM)

The in-vitro DPP-4 assay of the test extract shown 63.2% inhibition at 40 μg/mL. The positive control, sitagliptin, exhibited 91.7% inhibition (Fig. 1a, b).

### Serum DPP-4 activity assay

**Fig. 2:**
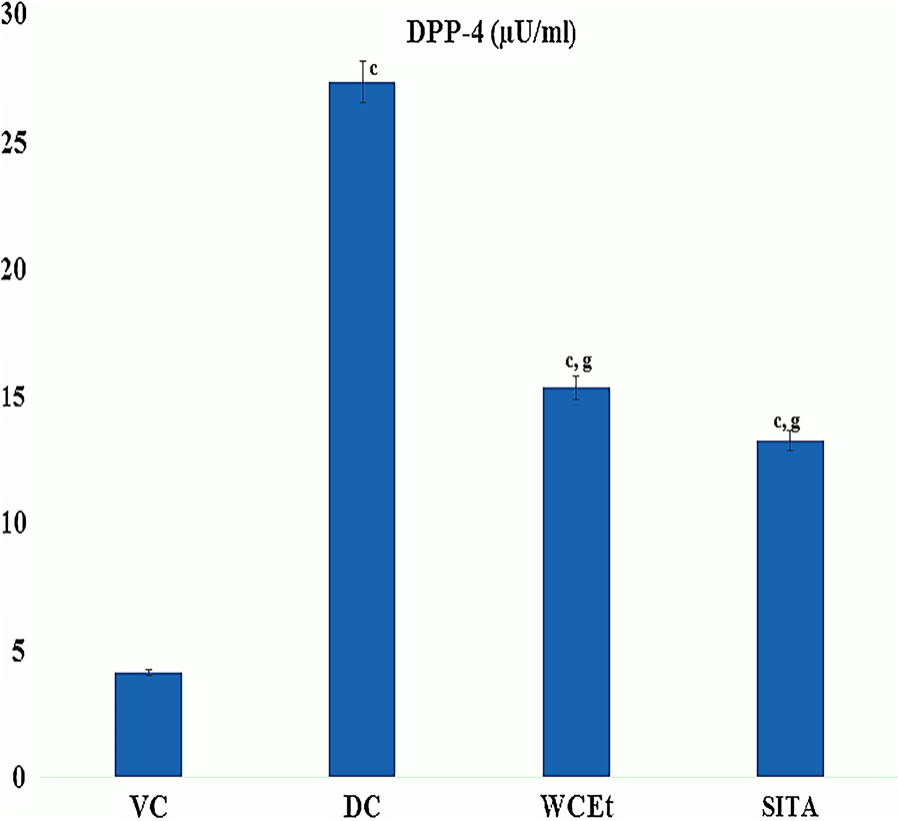
Serum DPP-4 levels in control and treatment groups. Data are means ± S.E.M. (*n* = 6); c, *P* ≤ 0.001; and d non-significant as compared to the respective control values.; g, *P* ≤ 0.001; and h non-significant as compared to the respective values of the diabetic control group

The serum levels of DPP-4 were significantly (*P* ≤ 0.001) elevated in type 2 diabetic control group (DR) in comparison to vehicle control animals. Whereas the treatments of the test extract and sitagliptin caused alterations in comparison to diabetic control and vehicle control (Fig. 2).

### LC–MS identification of the phytoconstituents present in an ethanol extract of *Withania coagulans* fruit

**Fig. 3:**
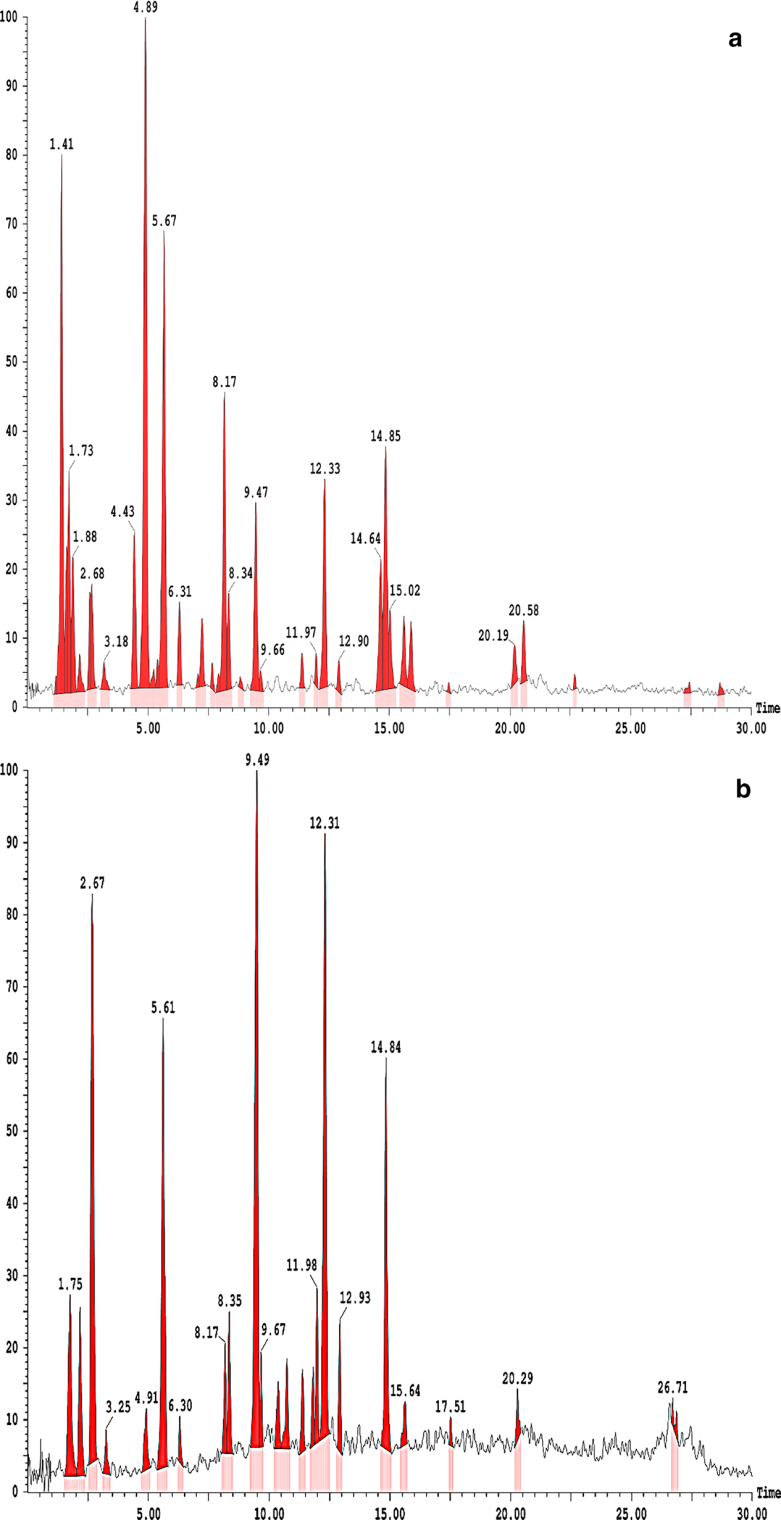
**a** QTOF of ethanol fruit extract of *Withania* *coagulans*. **b** QTOF of ethanol fruit extract of *Withania* *coagulans*

Several phytochemicals were detected in the positive mode of LC–MS analysis, including withanolide D, sitoindoside IX, withanoside IV, withanone, withanolide B, and withaferin A. Accordingly, the negative mode of LC–MS analysis identified four major compounds, withasomnine, withangulatin A, withacoagulin H, and withanolide E (Fig. 3a, b; Tables 1, 2).

**Table 1:** Identified masses from UPLC-QTOF mass spectroscopy constituents in the ethanolic fruit extract of *Withania* *coagulans* (Stocks) Dunal in positive electron ionization

S. no	Identified compound Name	Formula	Monoisotopic mass (g/mol)	Retention time (min)	m + z values
1	Withanolide D	C_28_H_38_O_6_	470.6	7.66	471.6
2	Sitoindoside IX	C_34_H_48_O_11_	632.7	8.83	633.7
3	Withanoside IV	C_40_H_62_O_15_	782.9	9.66	783.7
4	Withanone	C_28_H_37_O_6_	469.6	11.97	469.5
5	Withanolide B	C_28_H_38_O_5_	454.6	12.33	455.5
6	Withaferine A	C_28_H_38_O_6_	470.6	15.02	471.4

**Table 2:** Identified masses from UPLC-QTOF mass spectroscopy constituents in the ethanolic fruit extract of *Withania* *coagulans* (Stocks) Dunal in negative electron ionization

S. no	Identified compound Name	Formula	Monoisotopic mass (g/mol)	Retention time (min)	m − z values
1	Withasomnine	C_12_H_12_N_2_	184.24	7.11	183.2
2	Withangulatin A	C_30_H_38_O_8_	526.6	10.38	525.5
3	Withacoagulin H	C_28_H_36_O_6_	468.6	10.38	445.5
4	Withanolide E	C_28_H_38_O_7_	486.6	11.98	485.5

### Glucose homeostasis HOMA assessments of glucose homeostasis

**Fig. 4:**
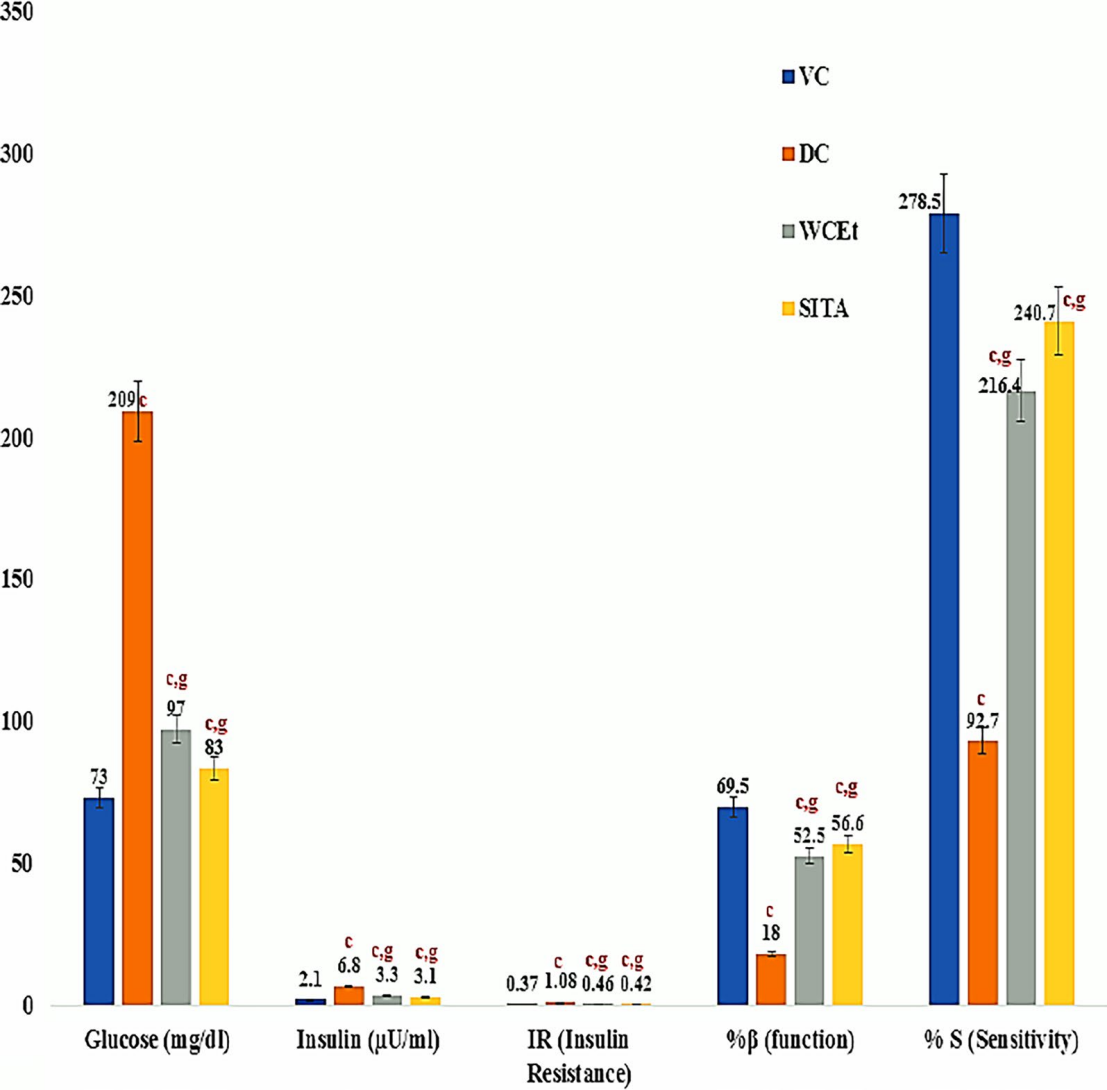
Effect of ethanol fruit extract of *Withania* *coagulans* on glucose homeostasis. Data are means ± S.E.M. (*n* = 6); c, *P* ≤ 0.001; and d non-significant as compared to the respective control values.; g, *P* ≤ 0.001; and h non-significant as compared to the respective values of the diabetic control group

Treatment of the type-2 diabetic rats with the test extract resulted in significant (*P* ≤ 0.001) beneficial alterations in glucose and insulin levels. Insulin resistance was significantly higher in the diabetic control group, while treatment with the fruit extract and sitagliptin resulted in a significant reduction in insulin resistance. Concomitantly, β- cell function and insulin sensitivity significantly increased in the fruit extract and sitagliptin treatment groups (Fig. 4).

### Alterations in the lipid profile

**Fig. 5:**
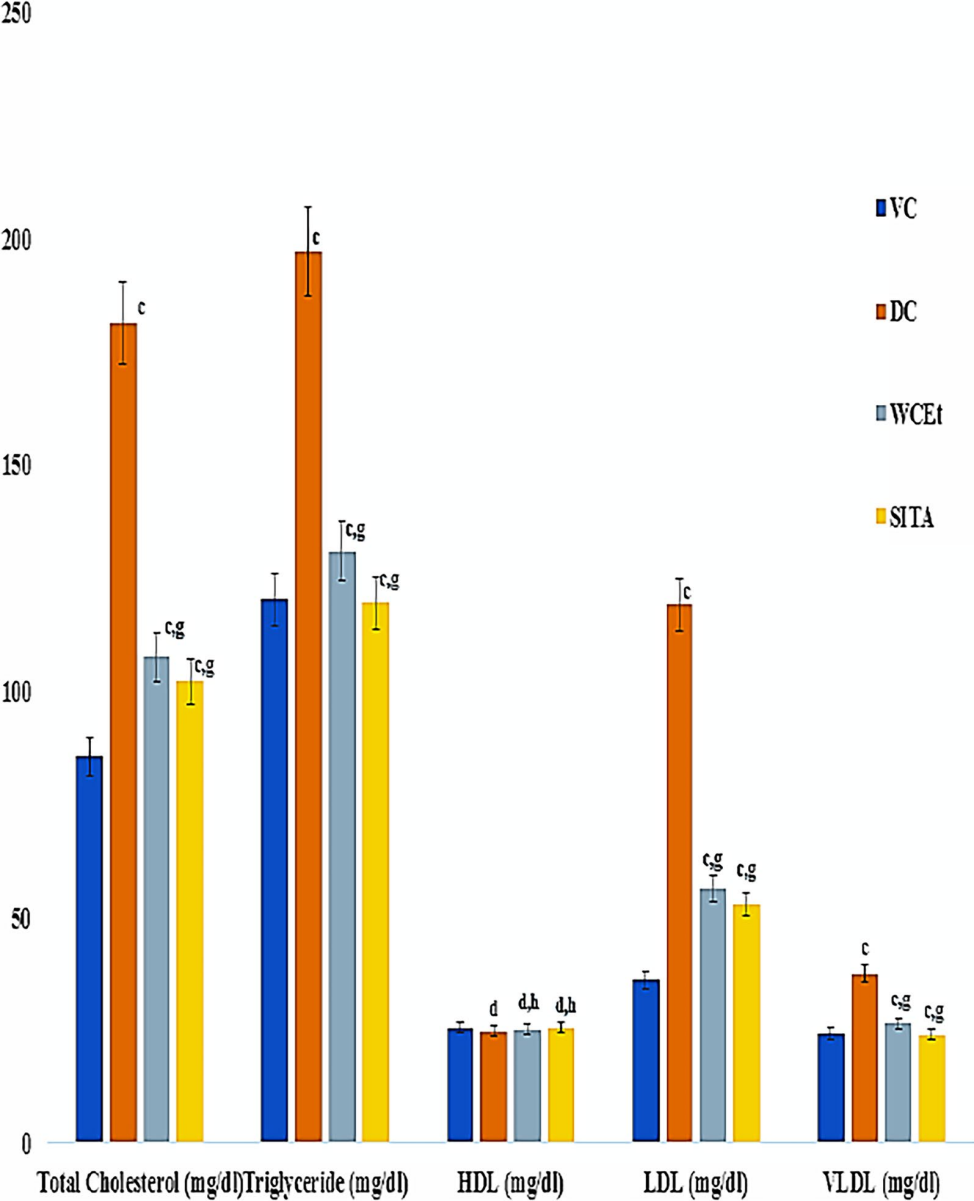
Effect of ethanol fruit extract of *Withania* *coagulans* on lipid profile. Data are means ± S.E.M. (*n* = 6); c, *P* ≤ 0.001; and d non-significant as compared to the respective control values. h Non-significant as compared to the respective values of the diabetic control group

Significantly (*P* ≤ 0.001) higher levels of total cholesterol, LDL-cholesterol, VLDL-cholesterol, and triglyceride, relative to the vehicle control and treatment groups, were observed in the diabetic control group. Treatment of the diabetic rats with the fruit extract resulted in a significant reduction in total cholesterol, LDL-cholesterol, VLDL-cholesterol, and triglyceride in comparison to the diabetic control group, as well as the sitagliptin-treatment group (Fig. 5).

### Antioxidants levels

**Fig. 6:**
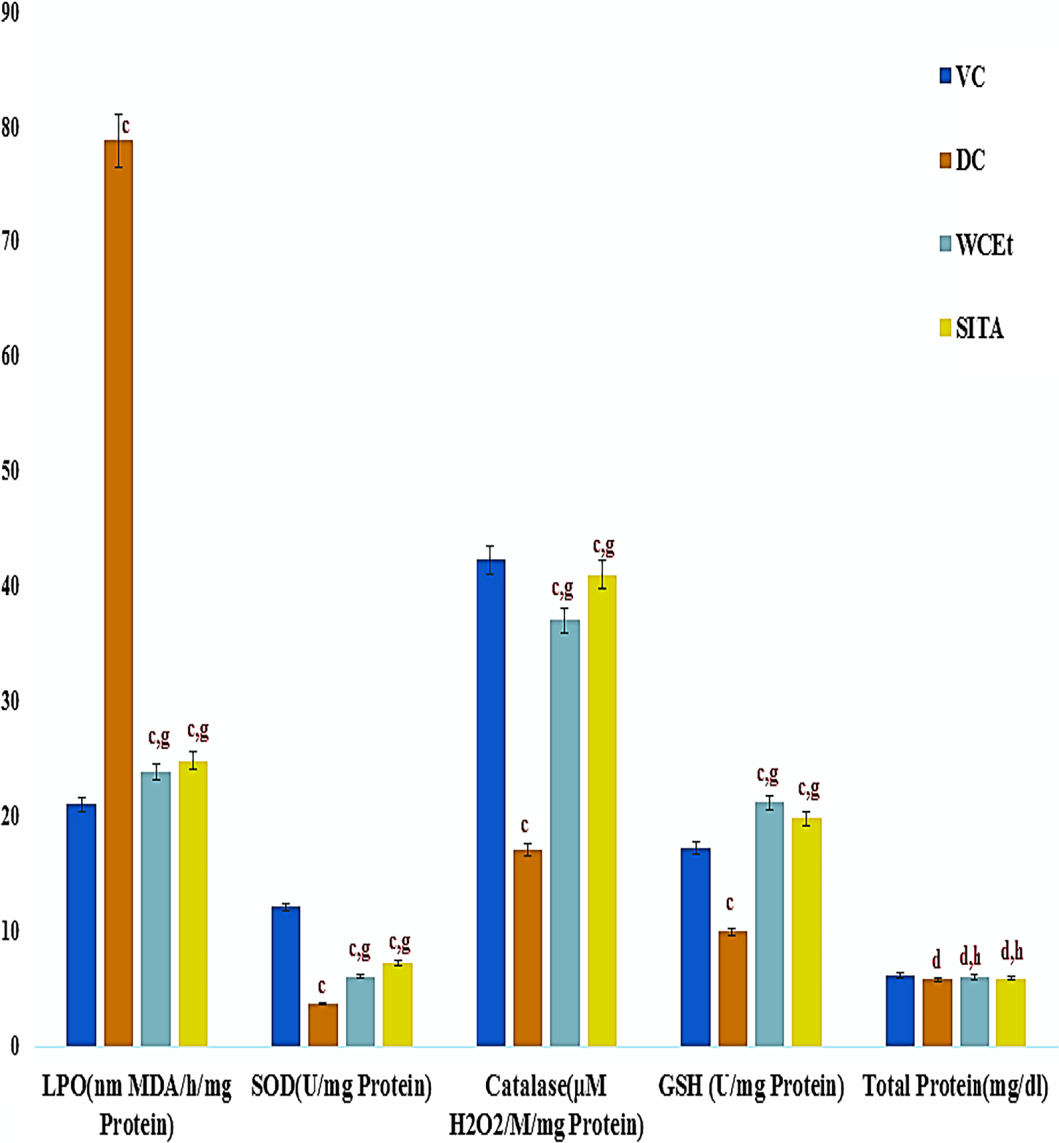
Effect of ethanol fruit extract of *Withania* *coagulans* Dunal on antioxidant levels. Data are means ± S.E.M. (*n* = 6); c, *P* ≤ 0.001; and d non-significant as compared to the respective control values. e, *P* ≤ 0.05; g, *P* ≤ 0.001; and h non-significant as compared to the respective values of the diabetic control group

The level of lipid peroxidation and total protein levels were significantly (*P* ≤ 0.001) higher in the diabetic control group, relative to the vehicle control group of animals, while the levels of catalase, GSH, and SOD were significantly reduced (*P* ≤ 0.001). The treatment of the diabetic animals with fruit extract resulted in a significant increase in the levels of GSH, SOD, and catalase, relative to the diabetic control group, as well as reduced levels of lipid peroxidation (Fig. 6).

### Histopathology of pancreas

**Fig. 7:**
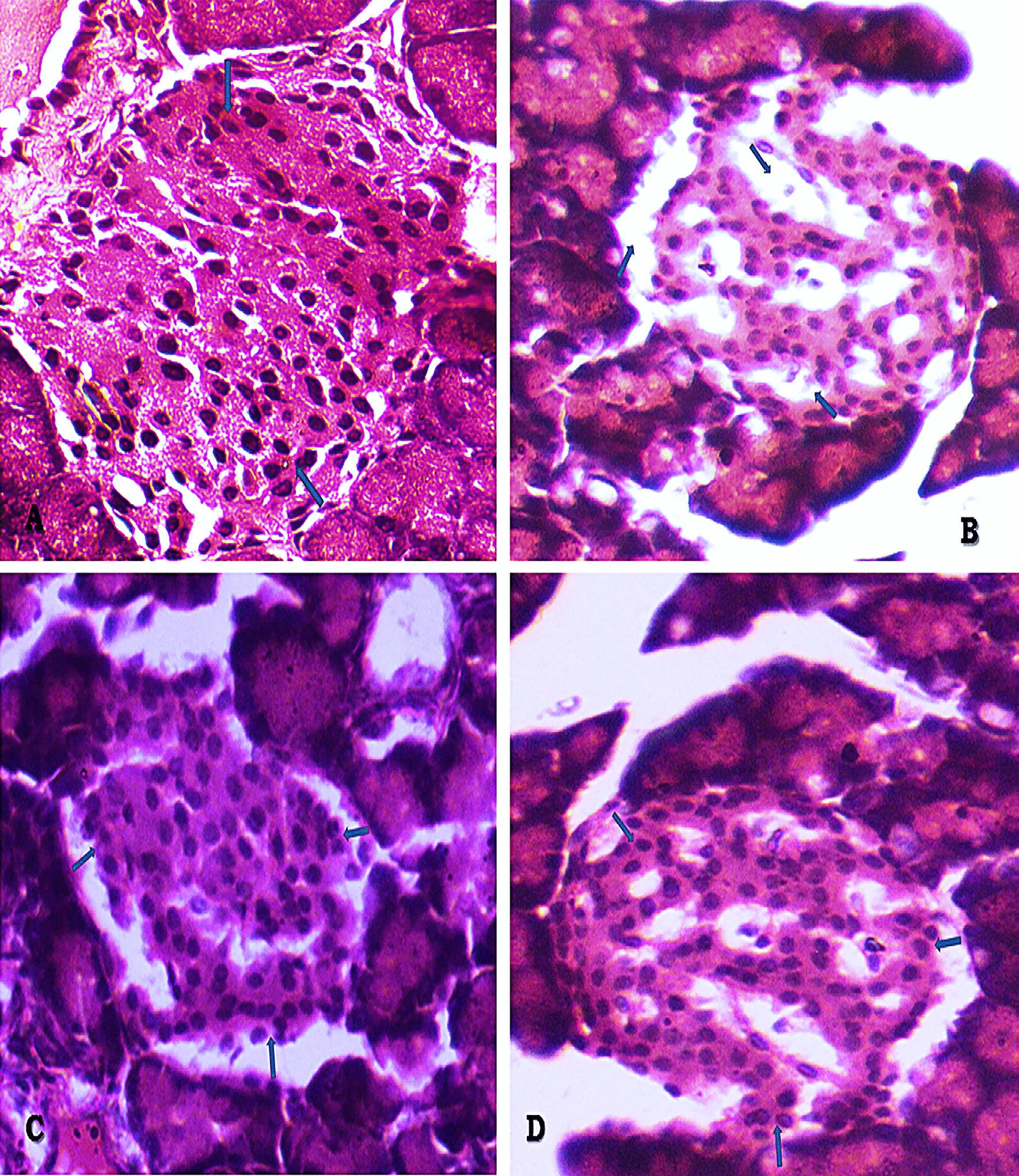
a–d Histology of different control and treatment groups (400X H&E): Arrows indicates the peripheral β-cell rich area with organised cellular mass of islet of the Langerhans (7A), degenerative area pointed by arrow (7B), arrow indicating the increased cellular mass (7C) and organized cellular mass pointed by arrow

Shrinkage and necrosis of the nuclei of islet cells and other degenerative symptoms were observed in pancreatic cells of the diabetic control group, relative to the vehicle control (Fig. 7a, b). In contrast, treatment of diabetic rats with the fruit extract resulted in a significant increase in islet cellular mass in pancreatic tissues, relative to the diabetic control and the sitagliptin-treatment groups. The treatment of the diabetic rats with either the fruit extract or sitagliptin also resulted in restoration of vascular tissues (Fig. 7c, d).

### In-silico molecular docking analysis

In-silico analysis of the small molecule phytochemicals of the teat extract and target protein made by following molecular docking (Protein-ligand) and ADME/T analyses. DPP-4 has a catalytic triad comprising Glu205, Glu206 and Tyr226 residues. The molecular interactions between the various identified phytoconstituents present in the fruit extract and the DPP-4 enzyme molecule were analyzed using AutoDock 4.2.6 software. Results indicated a variable degree of hydrogen bonding with the DPP-4 enzyme ranging from moderate to strong by the different phytochemicals present in the fruit extract. The identified compounds interacted with the main catalytic site residues with strong binding energies ranging from − 7.2 to − 9.8 (Kcal/mol); thus, inhibiting the protein irreversibly (Table 3).

**Table 3:** Molecular interactions of DPP-4 enzyme with detected compounds by LC–MS, present in ethanolic fruit extract of *Withania* *coagulans* (Stocks) Dunal

S. no	Ligand	Binding energy (Kcal/mol)	No. of H-bonds	Bond length (Å)	Interacting residues
*Positive control*					
1	Sitagliptin	− 8.9	2	3.3, 2.2	Glu205, Ser630
*Phytoconstituents*					
2	Withanolide D	− 9.2	1	2.1	Val207
3	Sitoindoside IX	− 9.8	4	2.4, 3.3, (3.2, 3.3)	Glu205, His740, Tyr547
4	Withanoside IV	Conformer generation is disallowed as too many atoms			
5	Withanone	− 7.9	4	(3.3, 3.4), 1.4, 2.3	Arg125, Tyr662, Val656
6	Withanolide B	− 9.5	2	3.1, 1.4	Tyr547, His740
7	Withaferine A	− 8.1	2	2.1, 2.4	Ser209
8	Withasomnine	− 6.6	1	2.5	Glu206
9	Withangulatin A	− 8.8	8	3.2, (3.2, 3.6), (3.2, 3.3) (3.2, 3.3), 3.2	Ser209, Arg125, Glu205, Glu206, Tyr662
10	Withacoagulin H	− 8.9	1	2.4, 2.9, 2.1, 2.3, 1.6	Glu206, Ser209, Tyr547, Glu205, Asp663
11	Withanolide E	− 7.6	4	2.8, 3.4, 3.4, 3.2	Glu206, Ser209, Asn710, His740

**Fig. 8:**
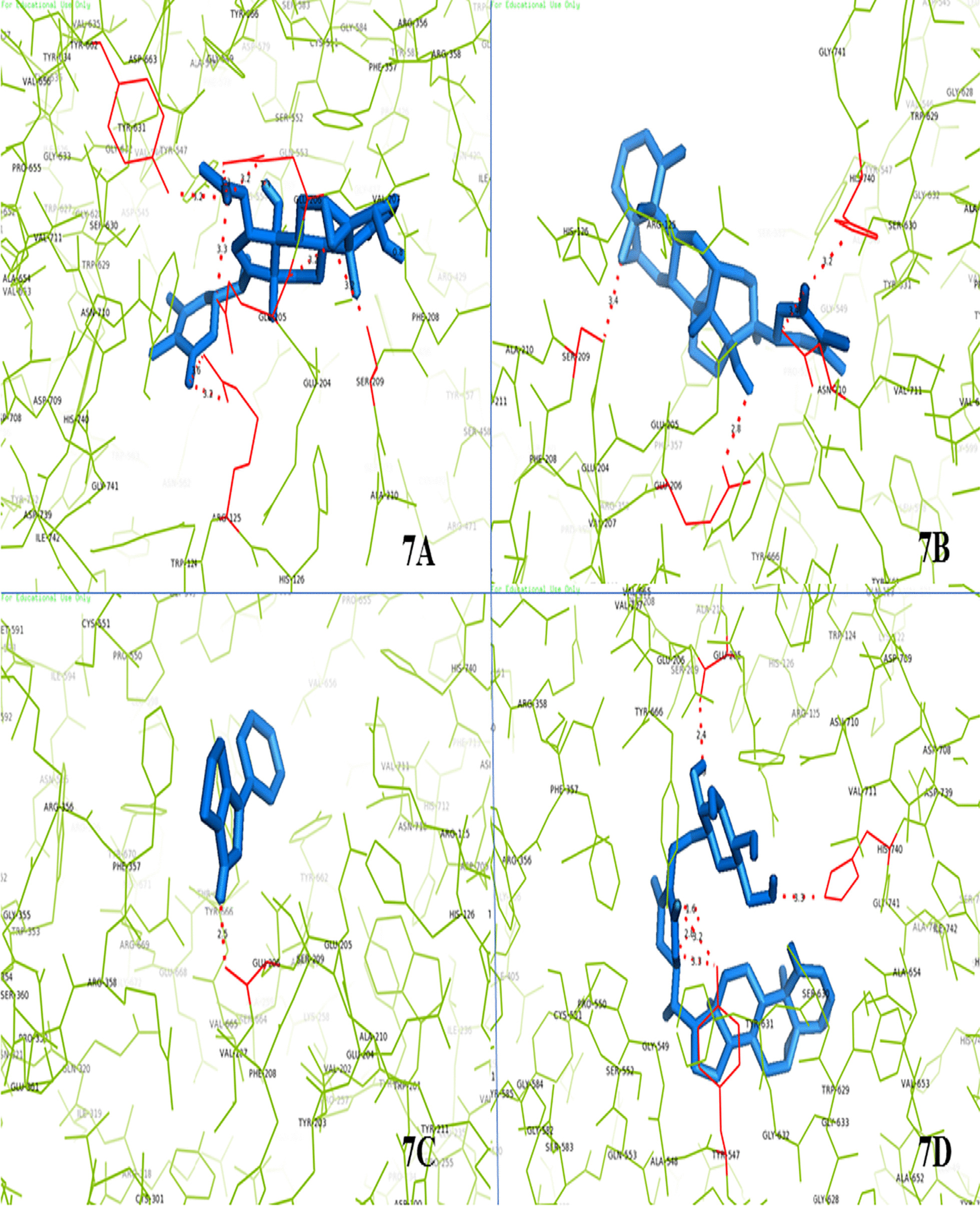

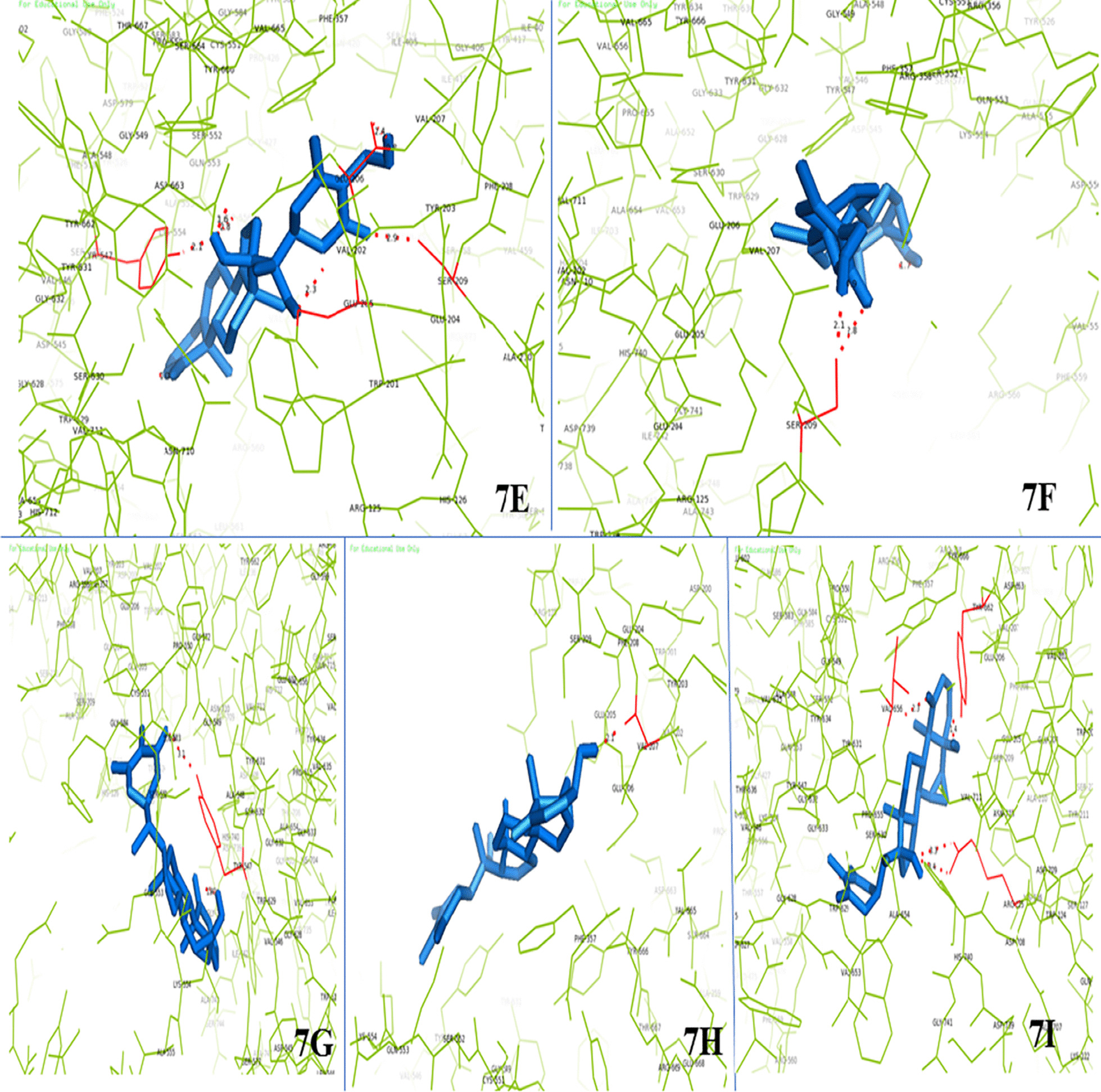
**a** DPP4 interaction with withangulatinA, **b** DPP4 interaction with withanolide E, **c** DPP4 interaction with withasomnine, **d** DPP4 interaction with sitoindoside IX. **e** DPP4 interaction with withacoagulin H; **f** DPP4 interaction with withaferine A; **g** DPP4 interaction with withanolide B; **h** DPP4 interaction with withanolide D; **i** DPP4 interaction with withanone

The phytochemicals present in the fruit extract exhibited stronger binding energies than the positive control (sitagliptin). Molecular interaction of the phytochemicals with the catalytic site residues by hydrogen bond formation was also detected in the molecular docking analysis (Fig. 8a–i).

### ADMET analysis

ADMET analysis of the identified phytoconstituents revealed that withasomnine was the only withanolide that met the Lipinski rule of five and had the potential to cross the BBB. Other withanolides and alkaloids met the Lipinski rule of an ideal drug but were determined to be unable to cross the BBB, which was most likely due to their large molecular size (Table 4). Sitoindoside IX and withangulatin-A violated the Lipinski rule of an ideal drug molecule and could not cross the BBB filter in the ADMET analysis.

**Table 4:** ADMET Pharmacokinetics of detected phytoconstituents of ethanolic fruit extract of *Withania* *coagulans* (Stocks) Dunal prediction by Drulito against Lipinski rule of five and blood–brain-barrier filter

Compound	MW	logP	AlogP	HBA	HBD	TPSA	nHB	nAcidic group	Filter L/B
Withanolide D	470.27	3.263	1.293	6	2	96.36	8	0	L
Sitoindoside IX	632.32	2.45	− 1.105	11	5	175.51	16	0	
Withanoside IV	102.07	1.311	− 0.73	2	1	37.3	3	1	L
Withanone	470.27	2.153	0.828	6	2	96.36	8	1	L
Withanolide B	454.27	4.118	1.539	5	1	76.13	6	0	L
Withaferine A	470.27	3.987	0.642	6	2	96.36	8	0	L
Withasomnine	184.1	2.436	0.991	2	0	15.6	2	0	L/B
Withangulatin A	526.26	1.126	0.685	8	2	122.66	10	0	
Withacoagulin H	468.25	1.903	1.021	6	3	104.06	9	0	L
Withanolide E	486.23	1.363	0.444	7	3	116.59	10	0	L

MW, molecular weight; logP, partition coefficient; AlogP, octanol–water partition coefficient; HBA, hydrogen bond acceptor; HBD, hydrogen bond donor; TPSA, total polar surface area; nHB, number of hydrogen bond; nAcidic group, number of acidic group; Filter L, Lipinski rule of five and B, blood brain barrier

## Discussion

The secretion of insulin regulated by postprandial stimulation and volume of the pancreatic β-cellular mass which is distressed by several mechanisms in type-2 diabetes [50]. The pancreatic β-cells are intricately controlled to constant activities and respond to nutrients, beneath the inflection of extra neurohormonal signals, in demand to secrete insulin to greatest encounter the requirements of the organism. The β-cell and nutrients recognizing involves multifaceted mechanisms of metabolic stimulation, ensuing in yield of stimulus-secretion linked signals that endorse insulin biosynthesis and release [50, 51]. In the current study, it was seen that high sucrose diet and corticosteroid caused insulin resistance and imbalanced glucose homeostasis which may following the several pathways and resulted in decreased β-cell mass and improper postprandial stimulations by degraded activities of GLP-1 [52]. The characterized hyperglycemia of diabetic condition is also causing glucotoxicity and lipotoxicity along with insulin resistance which further resulted in apoptosis of β-cells [53]. Whereas, the treatments of the test extract (*W. coagulans* fruit ethanol extract) and standard drug caused significant reductions in glucose, insulin and HOMA indices resulted in improvements in glucose homeostasis and increased pancreatic β-cell mass. These kinds of results may follow the interaction with DPP-4 by possesses active metabolites (phytochemicals) of extract through prolonging the GLP-1 postprandial stimulation to pancreatic tissues as reported by several studies [54, 55]. Accordingly, the results of LCMS analysis shown that occurrence of potent bioactive phytocompounds in ethanol fruit extract of *W coagulans* known as withanoids, including withanolide D, sitoindoside IX, withanoside IV, withanone, withanolide B, withaferin A, withasomnine, withangulatin A, withacoagulin H, and withanolide E. Subsequently, the in-vitro assay of the test extract against DPP-4 performed the 63.2% inhibition which validate the interaction with target enzyme (DPP-4) and phytocompounds. Accordingly, the serum DPP-4 activities were also increased after the treatments of test extract and sitagliptin.

In same context, it is illustrated that the phytocompounds have the ability to inhibit specific enzymes by binding to the active site within the enzyme molecule or a related mode of action [56, 57]. Ideal inhibitors have a low molecular weight that can reduce or completely inhibit enzyme activity at low concentrations [55]. Several human enzyme inhibitors, such as antithrombin and antitrypsin, control enzyme activity in the body, and can function under normal physiological conditions. Intermediary compounds are produced, however, by some natural enzyme inhibitors in some of the metabolic pathways. The inhibition of product formation is a way of controlling or modulating substrate flux through a metabolic pathway. If enzymes are sensitive to product inhibition, the output of the pathway will be suppressed [23, 58–61].

Administration of the fruit extract treatment to type-2 diabetic rats in the present study resulted in improved HOMA indices, as well as the restoration of normal histology in pancreatic tissues. Accordingly, the results resembles that phytochemicals have free radical scavenging capacity which may contribute to improvement in HOMA indices by reducing the generation and accumulation of free radicals [62, 63]. Our study demonstrated that the fruit extract and sitagliptin treatment resulted in significant changes in blood serum chemistry, including antioxidant potential. Reduced levels of free radicals may allow tissue regeneration to occur in the pancreas of the treatment groups.

Among the major phytochemicals identified in the fruit extract of *W. coagulans,* sitoindoside IX had thehighest binding energy (− 9.8 kcal/mol) to DPP-4, which was even higher than sitagliptin. These data suggest that this compound would have the greatest inhibitory activity against DPP-4. Binding energy is evidence of the degree of positive interaction that occurs between a target molecule, such as an enzyme, and the test compound or ligand. It is also a measure of the compatibility between a compound and its intended target [64, 65]. Sitoindoside IX and most of the other phytocompounds present in the fruit extract exhibited an ideal profile in the ADMET (Absorption, distribution, metabolism, excretion and toxicity) analysis, which indicates that the compound meets the five requirements of the Lipinski rule which is a measure of the bioavailability of a molecule and its ability to pass through the blood brain barrier [66, 67].

## Conclusion

Results indicated that the small molecule phytochemicals exhibited in an ethanol extract of *W. coagulans* fruit could inhibit DPP-4 and scavenge free radicals, resulting in an improvement in the HOMA indices as well as restoration in pancreatic tissues. Therefore, the study indicating the applications of small molecule phytoconstituents of the test extract for therapeutics of type-2 diabetes by validating the further studies with higher animal models and human subjects.

## Supplementary Information

**Additional file 1.** Composition of high sucrose diet for induction of type 2 diabetes animal model.

## Data Availability

All data used in this study has been included in this article.
