# Supplementary material for: Improvements in HOMA indices and pancreatic endocrinal tissues in type 2-diabetic rats by DPP-4 inhibition and antioxidant potential of an ethanol fruit extract of Withania coagulans
Source: Nutr Metab (Lond). 2021 Apr 21;18:43. doi: 10.1186/s12986-021-00547-2 (PMC8059290; doi:10.1186/s12986-021-00547-2)
Supplement: Supplementary file 1 — Additional file 1. Composition of high sucrose diet for induction of type 2 diabetes animal model. [file 12986_2021_547_MOESM1_ESM.docx]

**Composition of high sucrose diet for induction of type 2 diabetes animal model**

| **SN** | **Ingredients** | **Quantity (g/kg)** |
| --- | --- | --- |
| 1 | Casein | 200 |
| 2. | DL methionine | 3 |
| 3. | Corn Starch | 0 |
| 4. | Maltodextrin | 0 |
| 5. | Sucrose | 650 |
| 6. | Cellulose | 50 |
| 7. | Corn Oil | 50 |
| 8. | Salt Mix. | 35 |
| 9. | Vitamin Mix. | 10 |
| 10. | Cholin bitartrate | 2 |
| % of energy | | |
| 11. | Protein | 20 |
| 12. | Fat | 12 |
| 13. | Carbohydrate | 68 |

**Captions**

The diet formulation was made by following the incorporation of contents which promote the gluconeogenesis without interfere in feedback mechanism of carbohydrate metabolism as per previous studies [1–3].

**Reference**

1. Saifur Rohman M, Lukitasari M, Adi Nugroho D, Nashi W, Ida Panca Nugraheini N, Wahyu Sardjono eguh. Development of an Experimental Model of Metabolic Syndrome in Sprague Dawley Rat. Res J Life Sci. 2017;4:76–86.

2. Renee Commerford S, Ferniza JB, Bizeau ME, Thresher JS, Willis WT, Pagliassotti MJ. Diets enriched in sucrose or fat increase gluconeogenesis and G-6-Pase but not basal glucose production in rats. Am J Physiol - Endocrinol Metab. 2002;283:545–55.

3. Reeves PG, Nielsen FH, Fahey GC. AIN-93 purified diets for laboratory rodents: Final report of the American Institute of Nutrition ad hoc writing committee on the reformulation of the AIN-76A rodent diet. J Nutr. 1993;123:1939–51.

Dr. Heera Ram

(Corresponding Author)
